# Novel *ATM* Gene c.5644 C > T (p.Arg1882*) Variant Detected in a Patient with Pancreatic Adenocarcinoma and Two Primary Non-Small Cell Lung Adenocarcinomas: A Case Report

**DOI:** 10.3390/diseases10040115

**Published:** 2022-12-01

**Authors:** Abed A. Aljamal, Mohamad K. Elajami, Ephraim H. Mansour, Hisham F. Bahmad, Ana Maria Medina, Mike Cusnir

**Affiliations:** 1 Mount Sinai Medical Center, Department of Internal Medicine, Miami Beach, FL 33140, USA; 2 Department of Medicine, Division of Hematology Oncology, Medical University of South Carolina, Charleston, SC 29425, USA; 3 The Arkadi M. Rywlin M.D. Department of Pathology and Laboratory Medicine, Mount Sinai Medical Center, Miami Beach, FL 33140, USA; 4 Department of Translational Medicine, Herbert Wertheim College of Medicine, Florida International University, Miami, FL 33199, USA; 5 Mount Sinai Medical Center, Department of Internal Medicine, Division of Hematology and Oncology, Miami Beach, FL 33140, USA

**Keywords:** *ATM* gene, pancreatic adenocarcinoma, lung adenocarcinoma, case report

## Abstract

Ataxia-telangiectasia is an autosomal recessive disorder that usually manifests in childhood due to mutations in the Ataxia-Telangiectasia Mutated (*ATM*) gene. It is believed that there is an association between this gene mutation/polymorphism and cancer risk, including breast, lung, and pancreatic cancers. We report a rare case of a 69-year-old woman who developed three different primary cancers, including non-small cell lung cancer (NSCLC) in both lungs and pancreatic adenocarcinoma, and was later found to have a rarely reported variant mutation in the *ATM* gene, namely Exon 39, c.5644 C > T. We hypothesize that the *ATM* gene, c.5644 C > T mutation could be a plausible contributor in the pathogenesis of these three cancers. This hypothesis has yet to be validated by larger studies that focus on a mechanistic approach involving DNA repair genes such as the *ATM*. More importantly, this paves the way to developing new patient-specific targeted therapies and inaugurating precision medicine as a cornerstone in cancer therapeutics.

## 1. Introduction

Ataxia-telangiectasia is an autosomal recessive disorder that is caused by a defective Ataxia-Telangiectasia Mutated (*ATM*) gene [1]. Heterozygous mutations of the *ATM* gene have been associated with increased risk of developing malignancies through defective DNA-repairing mechanisms [2,3]. It is believed that there is an established association between *ATM* gene mutation/polymorphism and increased risk of breast cancer [4]. Studies have demonstrated that some variants of *ATM* are linked to an increased risk of breast cancer development and a worse prognosis [4,5]. Association with lung cancer, especially non-small cell lung cancer (NSCLC), and pancreatic cancer is becoming more established with many reported mutation variants in the literature for both cancers [6,7]. However, this relationship has yet to be fully understood, given the genetic complexity and the involvement of many other genes that harbor pathogenic variants in the pathogenesis of these types of cancers [8]. Over 1000 mutation variants, pathogenic and non-pathogenic, have been reported to involve the *ATM* gene [9].

In this case report, we discuss a rare case of a 69-year-old woman who developed three different primary cancers, including non-small cell lung cancer (adenocarcinoma) in both lungs and pancreatic adenocarcinoma. This patient was found to have a rarely reported variant mutation in the *ATM* gene, the Exon 39, c.5644C > T. This variant creates a premature translational stop signal (p.Arg1882*) that prompts loss of function in the *ATM* gene. Such mutation variants are known to be pathogenic. The c.5644C > T gene has been briefly mentioned in two studies by Coutinho et al. [2] and Buzin et al. [3]; this type of nonsense mutation causes a truncated, dysfunctional ATM protein. We hypothesize that the *ATM* gene c.5644C > T could be a plausible contributor in the pathogenesis of these three cancers. This case report was conducted and reported in accordance with the Surgical Case Reports (SCARE) guidelines for reporting case reports.

## 2. Case Presentation

**Figure 1:**
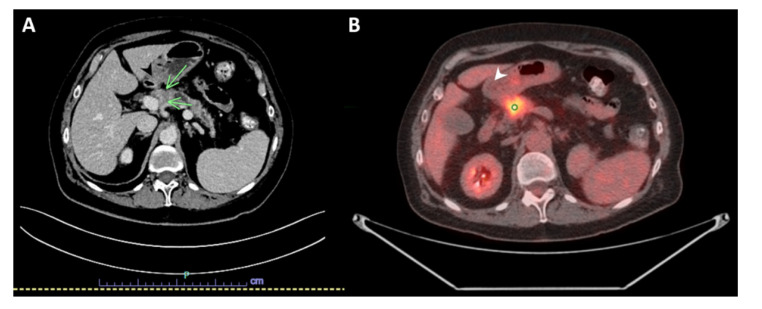
Imaging studies. (**A**) Computed tomography (CT) scan with IV contrast of the abdomen and pelvis showing a hypoenhancing pancreatic mass centered in the pancreatic neck, measuring 2.7 × 2 cm (green arrows). (**B**) Positron emission tomography (PET)/CT scan of the abdomen demonstrating mildly enlarged pancreas with a 2.7 cm heterogeneous, hypoechoic mass in the head of the pancreas with abnormal hypermetabolic uptake in the SUV MAX 9.3 (white arrow).

A 69-year-old woman with a history of primary left lung adenocarcinoma (confirmed and supported by immunohistochemical (IHC) stains) in 2014 that was treated with post left upper lobe resection and Cisplatin-based chemotherapy (treated at another institution), and contralateral primary lung adenocarcinoma in 2016 (confirmed and supported by IHC stains as well), which was treated at another institution with a right wedge resection, presented to our institution with abdominal discomfort. A computed tomography (CT) scan of the abdomen and pelvis with IV contrast demonstrated a hypoenhancing pancreatic mass centered in the pancreatic neck, measuring 2.7 cm in greatest dimension, with a dilatation of the upstream main pancreatic duct (up to 7 mm in diameter) and atrophy of the upstream pancreatic parenchyma that was consistent with pancreatic malignancy. The mass appeared to be abutting the splenoportal confluence with mild to moderate stenosis of the splenic vein, moderate stenosis of the superior mesenteric vein, and mild stenosis of the main portal vein at the confluence (Figure 1A). An abdominal positron emission tomography (PET)/CT scan was performed in April 2021, and showed a mildly enlarged pancreas with a 2.7 cm heterogeneous, hypoechoic mass in the head of the pancreas with abnormal hypermetabolic uptake in the SUV MAX 9.3. Multiple subcentimeter peripancreatic lymph nodes were seen without abnormal hypermetabolic uptake (Figure 1B).

**Figure 2:**
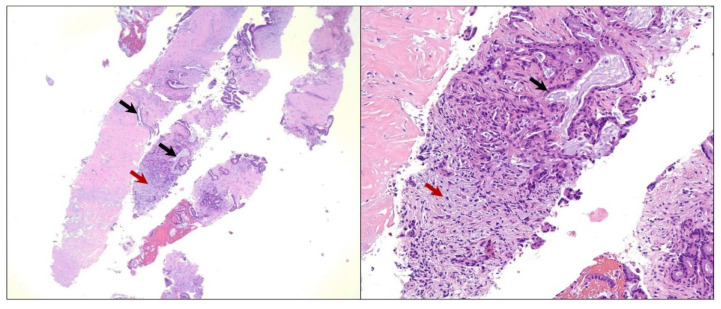
Microscopic image of the pancreatic fine needle aspiration specimen. Hematoxylin and eosin (H&E) staining showing neoplastic epithelial cells forming glands that are infiltrating the pancreatic stroma (black arrows). Also, a desmoplastic stromal reaction is seen (red arrows). The neoplastic cells show marked cytologic atypia, a high nuclear-cytoplasmic ratio, and nuclear pleomorphism, yielding a diagnosis of moderately differentiated adenocarcinoma in the head of the pancreas. Low-power magnification (40× objective) is shown in the panel to the left and high-power magnification (200× objective) is shown in the panel to the right.

The patient underwent an upper gastroesophageal endoscopy (EGD) with endoscopic ultrasound (EUS), showing an approximately 3 cm hypoechoic mass extending from the neck to the body of the pancreas. An EUS-guided fine needle aspiration (FNA) was performed at an outside institution, and histopathologic examination revealed neoplastic epithelial cells forming glands that were infiltrating the pancreatic stroma (Figure 2). The neoplastic cells showed marked cytologic atypia, a high nuclear-cytoplasmic ratio, and nuclear pleomorphism. A diagnosis of primary moderately differentiated adenocarcinoma in the head of the pancreas was made.

Initial laboratory results, including complete blood count, chemistry profile, liver enzymes, and liver function tests, were within normal reference ranges. The cancer antigen 125 (CA-125) was normal at 12 u/mL (standard range 0.0–38.1 u/mL), the carbohydrate antigen 19-9 (CA19-9) was elevated at 257 u/mL (standard range 0–35 u/mL), and the carcinoembryonic antigen (CEA) was borderline elevated at 4.9 ng/mL (standard range 0–4.7 ng/mL).

Genetic analysis testing of the patient’s blood was performed through an INVITAE multi-cancer panel (Invitae Corp., San Francisco, CA, USA). The Invitae Multi-Cancer Panel analyzes 84 genes associated with genetic disorders and hereditary cancers across major organ systems, including: breast and gynecologic (breast, ovarian, uterine), gastrointestinal (colorectal, gastric, pancreatic), endocrine (thyroid, paraganglioma/pheochromocytoma, parathyroid, pituitary), genitourinary (renal/urinary tract, prostate), skin (melanoma, basal cell carcinoma), brain/nervous system, sarcoma, and hematologic (myelodysplastic syndrome/leukemia). Results revealed one pathogenic variant identified in the *ATM* gene. This gene is associated with autosomal dominant predisposition to certain cancers and autosomal recessive ataxia-telangiectasia. The results are summarized in Table 1, showing mutation in the *ATM* gene Exon 39, c.5644C > T (p.Arg1882*) variant. 

In addition, Guardant360^®^ CDx testing (Guardant Health, Redwood City, CA, USA) was performed on the patient’s blood to provide tumor mutation profiling for her advanced solid malignancies. Guardant360^®^ CDx sequences 74 cancer-associated genes to identify somatic alterations. Cell-free DNA (cDNA) is extracted from plasma, enriched for targeted regions, and sequenced using the Illumina platform and hg19 as the reference genome. All exons are sequenced in some genes; only clinically significant exons are sequenced in other genes. The types of genomic alterations detected by Guardant360 include single nucleotide variants, gene amplifications, fusions, short insertions/deletions (the longest detected was 70 base pairs), and splice site disrupting events. Moreover, microsatellite instability (MS) is assessed for all cancer types by evaluating somatic changes in the length of repetitive sequences on the Guardant360 panel. Guardant360^®^ CDx is a qualitative next generation sequencing (NGS)-based in vitro diagnostic device that uses targeted high throughput hybridization-based capture technology for the detection of single nucleotide variants (SNVs), insertions and deletions (indels) in 55 genes, copy number amplifications (CNAs) in 2 genes, and fusions in 4 genes. Guardant360 CDx utilizes circulating cell-free DNA (cDNA) from the plasma of peripheral whole blood collected in Streck Cell-Free DNA Blood Collection Tubes (BCTs). The test is intended to be used as a companion diagnostic to identify non-small cell lung cancer (NSCLC) patients who may benefit from treatment with the targeted therapy in accordance with the approved therapeutic product labeling. The patient’s results revealed two detected somatic alterations, as shown in Table 2, one of which was the *ATM* gene (R1882* alteration), which was consistent with INVITAE multi-cancer panel’s results.

**Figure 3:**
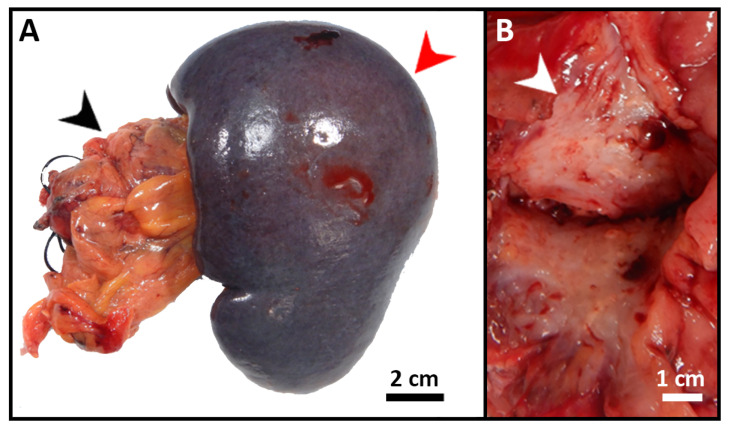
Gross images of the resected pancreatic tumor. (**A**) Segment of distal pancreas (black arrow) with attached spleen (red arrow) and attached hilar adipose tissue. (**B**) Cut section through the tail of pancreas revealed a 3 cm firm mass (white arrow) with a white-to-yellow indurated cut surface.

**Figure 4:**
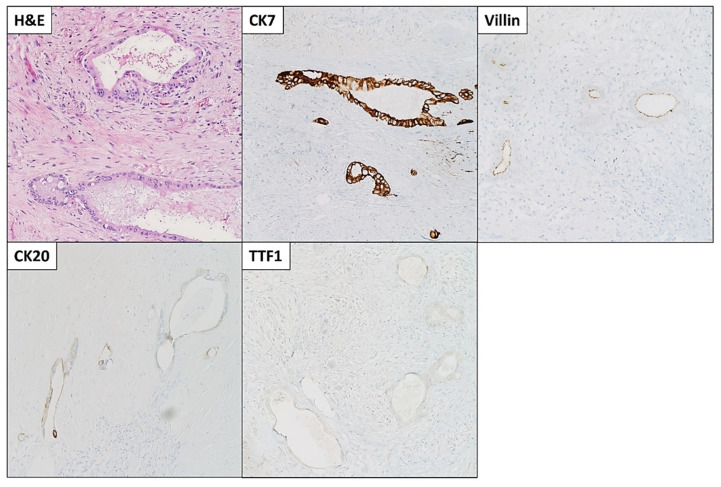
Microscopic images and immunohistochemical (IHC) stains of the pancreatic moderately differentiated ductal adenocarcinoma. Tumor cells were positive for cytokeratin (CK)7, CK20 (weak), and villin. TTF1 was negative. Microscopic images were examined at 200× objective.

The patient was started on FOLFIRINOX (Leucovorin, 5-Fluorouracil (5-FU), Irinotecan, and Oxaliplatin) as a neoadjuvant therapy and completed six cycles. She also received a total of 45 Gray (Gy) adjuvant radiotherapies. After chemoradiation, she was referred to surgical oncology and underwent staging laparoscopy, extended distal pancreatectomy and splenectomy, distal gastrectomy, left hepatectomy and cholecystectomy with patch repair of portal vein, and celiac to right hepatic arterial bypass (Figure 3A). A 3 cm firm mass with a white-to-yellow indurated cut surface was found extending throughout the body and tail of the pancreas (Figure 3B). Histopathologic examination confirmed the biopsy’s diagnosis of pancreatic moderately differentiated ductal adenocarcinoma. Immunohistochemical (IHC) stains were positive for cytokeratin (CK)7, CK20 (weak), and villin. TTF-1 was negative, which supported the diagnosis (Figure 4).

The tumor was confined to the pancreas with negative surgical margins. The treatment effect was evident where extensive parenchymal atrophy with associated fibrosis were appreciated. Residual cancer with evident tumor regression was seen, but more than single cells or rare small groups of cancer cells, which are referred to as partial response (score 2) according to the College of American Pathologists (CAP) cancer protocol. All 32 excised lymph nodes were negative for carcinoma (0/32). Also, the splenectomy, distal gastrectomy, left hepatectomy, and cholecystectomy specimens were free of carcinoma. Therefore, the pTNM pathologic stage (American Joint Committee on Cancer (AJCC) 8th Edition) given was ypT2N0M0.

The patient’s postoperative course was complicated by duodenal stump leak and sepsis, for which the patient had a washout. The patient had a worsening liver ischemia, due to hepatic artery thrombosis and pneumonia, with subsequent respiratory failure that required intubation. She ultimately expired 11 days after the surgery.

## 3. Discussion

The *ATM* gene is located on human chromosome 11q22-q23 and consists of 66 exons that span 150 kb of genomic DNA [10]. It encodes a PI3K-related serine/threonine protein kinase that plays a pivotal role in double-strand DNA break repairs. Genes that are involved in repairing double-strand DNA breaks, such as *BRCA1/2*, *ATM*, *PALB2*, *CHEK1/2*, *RAD51*, and *ATR*, can sometimes carry germline or somatic mutations that hinder the normal function of these genes [11,12].

As a master regulator of DNA damage and repair, the *ATM* gene is responsible for both homologous repair (HR) and non-homologous end joining (NHEJ) [13]. Either a germline or an induced mutation (i.e., radiotherapy induced) can alter the function of the *ATM* gene initiating carcinogenesis [14].

The *ATM* gene is associated with autosomal dominant predisposition to breast, pancreatic [15], and possibly prostate cancer [16,17], in addition to autosomal recessive ataxia-telangiectasia (A-T). There is also preliminary evidence suggesting that *ATM* is associated with autosomal dominant predisposition to other cancer types, including stomach [18], ovarian [19], bladder [20], and colon [21]. The pathogenic variant of the *ATM* gene identified in our patient has been observed in individuals with ataxia-telangiectasia and breast cancer [2,3,22,23,24]. Importantly, the *ATM* R1882* (c.5644C > T) alteration that was detected in our patient’s sample was at an allele fraction that was suspicious for its germline origin. This variant may lead to the loss of functional protein, and similar variants have been associated with hereditary predisposition to cancer. However, the germline versus somatic origin of this finding cannot be confirmed.

The *JAK2* V617F mutation in the patient’s sample was an extremely rare mutation in the patient’s stated diagnosis, but is very common in myeloproliferative neoplasms such as polycythemia vera (>95% of cases) and essential thrombocythemia (approximately 50% of cases), which could raise the possibility of an incidental finding of a second myeloproliferative neoplasm [25,26]. However, the patient’s normal complete blood count, with differential and lacking signs and symptoms of a myeloproliferative neoplasm, makes this possibility unlikely.

In our case report, we hypothesize that this rarely reported variant mutation in the *ATM* gene is possibly linked to the pathogenesis of both NSCLC and pancreatic cancer. The *ATM* gene is highly polymorphic, and mutations within this gene increase the risk of breast cancer. Over the past 10 years, it became more evident that pancreatic cancer and lung cancer are also associated with pathogenic *ATM* mutations [6,7]. This presumable association is possibly related to the advancement in gene analysis and next-generation sequencing (NGS) that is allowing more gene variants to be discovered in the appropriate clinical setting.

Multiple studies demonstrated the relationship between pancreatic cancer and pathogenic *ATM* gene variants [7,11]. A study by Klavanian et al. revealed an association between harboring pathogenic *ATM* gene mutations and increased family history of pancreatic cancers [27]. The study showed that among 114 patients with identified *ATM* mutations, 22 of them (19.3%) had a family history of pancreatic cancer. Besides, two of the tested families had the high-penetrance *ATM* mutation c.7271T > C (p.V2424G) [27]. Furthermore, Roberts et al. found that four variants of the *ATM* genes c.3214G > GT; p.E1072X, c.6095G > GA; p.R2032K, IVS41-1G > GT, and c.3801delG were identified in 166 pancreatic cancer patients, whereas these variants were not identified in the control group [28].

Heterozygous *ATM* mutations can be associated with breast cancer, especially the missense mutation *ATM* c.7271T > G (p.Val2424Gly) [12]. Whether these mutations are associated with pancreatic cancer or not has yet to be determined. However, we are adding here a new mutation variant that could be linked to the pathogenesis of both lung and pancreatic cancer. To the best of our knowledge, there are no studies delineating an association between the presence of the mutation variant we discovered in our patient and the occurrence of pancreatic cancer.

Besides the newly discovered pancreatic cancer, our patient had a history of primary non-small cell lung cancer in both lungs. The lungs are the most commonly exposed organ to environmental DNA-damaging agents, and more research is showing that genetic heterogeneity (either somatic or germline) is implicated in the pathogenesis of lung cancer [29,30,31]. Among the various DNA damage response and repair (DDR) genes, the *ATM* gene remains one of the leading genes in that sphere, but that comes at a cost of harboring many somatic and germline mutations. Up to 34% of patients with pancreatic ductal adenocarcinoma have germline mutations in the *ATM* gene [7]. The tumor suppressor gene *TP53* and proto-oncogenes, such as *KRAS*, *EGFR*, and *ALK*, are well studied in the pathogenesis of lung cancer [32,33,34,35]. In addition to those genes, more studies are demonstrating a correlation between homologous repair (HR) genes, including *BRCA1*, *BRCA2* and *ATM*, with non-small cell lung cancer [36].

Based on a study by Scheffler et al., 11.9% of 1078 lung cancer patients with *KRAS* mutations had concomitant mutations in 12 different locations in the *ATM* gene [37]. This suggests a role for the latter gene in lung cancer. Henceforth, *ATM* gene mutations are presumably linked to both pancreatic and lung cancer, especially NSCLC. Nevertheless, it is still unclear whether the *ATM* c.5644C > T variant is indeed linked to the pathogenesis of the aforementioned cancers that our patient had. More research is warranted to decipher the exact role of this gene mutation variant in this cancer association.

## 4. Conclusions

In the setting of complex disorders such as pancreatic and lung cancers, each of which harbor various genetic aberrations and molecular signature mutations, common hits could instigate a role in the pathogenesis of each cancer. The fact that more than one cancer occurred concomitantly in our patient raises the question whether a single nonsense mutation might have driven a carcinogenic pathway of those cancers, this being the *ATM* c.5644C > T variant in our case. Indeed, our hypothesis has yet to be validated by larger studies that focus on a mechanistic approach involving DNA repair genes such as the *ATM*. More importantly, this paves the way to developing new patient-specific targeted therapies and inaugurating precision medicine as a cornerstone in cancer therapeutics.

## Figures and Tables

**Table 1:** Results of the genetic analysis testing of the patient’s blood performed through INVITAE multi-cancer panel.

Gene	Variant	Zygosity	Variant Classification
*ATM*	c.5644C > T (p.Arg1882*)	Heterozygous	Pathogenic

**Table 2:** Results of the genetic analysis testing of the patient’s blood performed through Guardant360^®^ CDx testing.

Detected Alteration(s)/Biomarker(s)	Associated FDA-Approved Therapies	Clinical Trial Availability	% cfDNA or Amplification
*ATM* R1882*	Niraparib, Olaparib, Rucaparib, Talazoparib	Yes	43.6%
*JAK2* V617F	Ruxolitinib	Yes	7.4%

## Data Availability

Not applicable.
